# Editorial: Planetary health impacts of pandemic coronaviruses

**DOI:** 10.3389/fpubh.2022.987167

**Published:** 2022-08-09

**Authors:** David López-Carr, Susanne Sokolow, Giulio De Leo, Kris Murray, Michele Barry

**Affiliations:** ^1^Department of Geography, University of California, Santa Barbara, Santa Barbara, CA, United States; ^2^Hopkins Marine Station, Stanford University, Stanford, CA, United States; ^3^Hopkins Marine Station, Imperial College London, London, United Kingdom

**Keywords:** planetary health, coronavirus (COVID-19), public health, infectious disease, prevention

Novel pandemic coronavirus (SARS CoV-2) originated in Asia in late 2019 and has spread rapidly and indiscriminately worldwide. At the time of publication, the virus has caused over 553 million confirmed cases and over 6.3 million deaths globally, resulting in the most devastating pandemic since the Spanish flu in 1918. A distinct feature of the COVID-19 pandemic is that its full range of impacts far exceeds those resulting from the disease itself. No country in the world has been spared by the socio-economic devastation caused by the pandemic lockdown and there is still not yet a full understanding of the direct and indirect implications for human health and wellbeing in the near and longer term. Subsequent collateral impacts on the natural world, as well as our ultimate goals to live sustainably within it, can therefore also be expected. At the same time, lessons and opportunities that could help catalyze change toward a more sustainable, resilient, and healthy future can be identified. With nature itself being the largest source of pathogens that can potentially spillover from their animal reservoirs and affect human health, such wide ranging, intersectoral, and potentially transformational impacts make pandemic emergencies of central relevance to the emerging field of planetary health.

We invited submissions of original research, mini reviews, and perspectives on the planetary health impacts and opportunities of pandemic emergencies, including but not restricted to COVID-19. Contributions drawing key insights from past pandemics were welcome as were articles making novel contributions to the field of future pandemic preparedness, risk assessment and management. Submissions focusing on non-human outbreaks that offer the potential for cross-disciplinary impact were also considered. Ultimately, contributions to the special issue generally fit under two categories: The climate and nature dynamics of pandemic coronaviruses and challenges and potential solutions surrounding public health interventions.

Regarding climate and nature dynamics, the issue published five contributions. Heibati et al. consider how weather might influence the incidence of COVID-19 infection. The authors employed a quasi-Poisson generalized additional model to examine the potential role of various meteorological factors on daily counts of COVID-19 in Finland during several months in 2020. The authors found no associations between daily temperature and COVID-19 incidence. However, daily average relative humidity was negatively related to COVID-19 rates in two hospital districts although no relation was found at the national scale. The authors conclude that there was no statistically significant relation between meteorological variables and COVID-19 incidence at least in the Finnish study context of arctic and subarctic winter and spring. However, given the small period and modest number of cases, future research on the topic is recommended.

In studying the potential connection between people's exposure to nature due to the COVID-19 outbreak, Lenaerts et al. note that confinement measures to reduce viral spread was implemented globally and, as a result, there was an increase in people exercising outdoors. The authors conducted a survey to assess the extent to which people might visit nature and specifically if these visits might have increased in frequency following restrictions to minimize infectious transmission. Based on 11,352 survey participants in Flanders, Belgium, bivariate and multiple regression results suggest that people indeed have visited nature more frequently than before restrictions and furthermore that nature assisted in sustaining social relationships during a time of coronavirus restrictions.

Codeco et al. note that the Amazon ecosystem is threatened by increasing deforestation and biodiversity loss while also maintaining a high level of tropical diseases. The authors research the relative distribution of six archetypal development trajectories in relation to vulnerability to tropical diseases and environmental degradation. The team finds that small farmer trajectories represent approximately half of the Amazon territory, especially in areas where malaria is rife. Along with the dominant peasant development trajectories, cattle (associated with increased deforestation) and large-scale farm and livestock producing trajectories were associated with a high prevalence of neglected tropical diseases, such as leishmaniasis, *Aedes*-borne diseases and Chagas disease along with biodiversity loss. These results show how land-use change and biodiversity loss driven by agricultural expansion and intensification is often associated to undesired and in some cases unexpected negative outcomes for human health.

Kalema-Zikusoka et al. also work in a tropical forest environment and research potential links between COVID-19 and the health and conservation of endangered mountain gorillas. They provide the example of reduced tourism income leading to increased poaching and ultimately the killing of a gorilla at the hands of a hungry community member. Conservation Through Public Health (CTPH), an NGO that promotes biodiversity conservation, animal and human health and livelihoods in the area of Africa's protected areas along with the Uganda Wildlife Authority have taken steps to improve great ape viewing while preventing COVID-19 transmission between people and gorillas. Behaviors to decrease transmission included the use of face masks, improved hand hygiene, and a 10-meter great ape viewing distance.

Rounding out the nature-infectious disease collection of articles in the special issue, Nova reviews the state of knowledge regarding cross-species transmission of coronaviruses in humans and domestic mammals. She finds that several novel coronaviruses have emerged in humans, domestic and wild animals during the last several decades and has been facilitated by cross-species transmission. She further finds that the coronaviruses were closely related and likely associated with high-host-density environments that facilitate multi-species interactions. She concludes with a call for further research on cross-species transmission, especially in the context of increasing environmental change and degradation.

Public health interventions included six papers in the special issue. Himmel and Frey examine controversial biological and political issues surrounding COVID-19. The authors make various recommendations for actions under the rubric of the World Health Organization and the Biological Weapons Convention.

Rakotonanahary et al. respond to the question of how to control the global COVID-19 pandemic, especially in Africa. The authors argue that the primary challenge in responding to COVID-19 is the integration of several health areas including ‘'prevention, testing, front line health care, and reliable data to inform policies.” The team presents a COVID-19 strategy in Ifanadiana District with the Malagasy Ministry of Public Health and non-governmental organizations as partners. The authors describe the contours and challenges of their integrated response and how various data sources can be used to address the science of COVID-19. Despite a second COVID-19 wave in March 2021, results showed fewer cases in Ifanadiana than for many other diseases (e.g., malaria).

Baker et al. argue that the COVID-19 pandemic has exposed the inadequacy of the U.S. healthcare system, which was exacerbated by the estimated $202 billion loss for the healthcare industry from the disease. They argue that while the demand for personal protective equipment (PPE) grows, more sustainable solutions will be necessary to reduce supply, cost, and waste challenges. As a proposed solution, the authors examine the advantages of reusable gowns. Among reusable gown advantages, polyester material reduces microbial cross-transmission, hospitals report a 50% lower cost than with disposable gowns, and reusable gowns reduce energy and water use as they can last through 75–100 launderings compared to single-use disposable gowns.

Huntigford et al. confront the important issue of vaccine justice. Using a Susceptible-Infected-Recovered-Vaccinated (SIRV) compartmental model, the authors simulate COVID-19 dynamics within and between two countries. Nation one produces a vaccine and decides how it may be shared with nation two. Overlapping with the authors' mathematical structure is the effect of travel between the two nations during a pandemic. Results show that, even when taking into account substantial travel between the two nations, nation one minimizes its total mortality by retaining vaccines and aspiring for full inoculation as quickly as possible. This result suggests that travel risks can be reduced by a fast vaccination campaign. The authors find also that, while a country is better off when it maximizes its own vaccination rate, the total number of COVID-19 associated deaths can be minimized only when vaccine-producing countries share vaccines with countries lacking the capacity to produce one. This raises important political and ethical questions regarding vaccine sharing between wealthier and poorer nations.

Pan et al. estimate national and sub-national effect sizes of non-pharmaceutical interventions (NPI) to control COVID-19 during the initial months of the US pandemic. A problem in such endeavors to date is that effect size estimates ‘'have not accounted for heterogeneity in social or environmental factors that may influence NPI effectiveness” according to the authors. Using daily county-level COVID-19 cases and deaths, doubling times and mortality rates were compared to ‘'four increasingly restrictive NPI levels.” Using a ‘'stepped-wedge cluster-randomized trial analysis” results suggest that ‘'aggressive (level 4) NPIs were associated with slower COVID-19 propagation” and a longer duration of level 4 NPIs was related to lower case rates and longer doubling times. They also found heterogeneity in NPI effectiveness across US Census regions which suggests that control strategies may be most optimally designed at the community-level.

Completing the special issue, Fendt et al. examine the demand for face masks in Germany. They note that non-reusable masks are often incorrectly disposed and are not biodegradable, increasing their environmental impact. The authors question, however, to what extent mask users are conscious of this, and the factors that may impact face mask choice. Investigating ‘'user preferences, perceived effectiveness, and the sustainability of different mouth/nose protection (MNP),” the authors use a national sample of 1,036 participants to describe trends among respondents. Results suggest that protective effectiveness, and the reusability of MNP are important to most respondents and especially to older informants. Conversely, ‘'the price, shape, and design were not as important.” The authors conclude that there appears to be a preference for sustainable MNP so long as their protection remains equivalent to medical or FFP2/FFP3 masks.

## Author contributions

DL-C drafted the bulk of the paper. SS and GD wrote parts of the paper. All authors contributed to the conceptualization and editing of the paper.

## Conflict of interest

The authors declare that the research was conducted in the absence of any commercial or financial relationships that could be construed as a potential conflict of interest.

## Publisher's note

All claims expressed in this article are solely those of the authors and do not necessarily represent those of their affiliated organizations, or those of the publisher, the editors and the reviewers. Any product that may be evaluated in this article, or claim that may be made by its manufacturer, is not guaranteed or endorsed by the publisher.

